# Conditional genetic deletion of CSF1 receptor in microglia ameliorates the physiopathology of Alzheimer’s disease

**DOI:** 10.1186/s13195-020-00747-7

**Published:** 2021-01-05

**Authors:** Vincent Pons, Pascal Lévesque, Marie-Michèle Plante, Serge Rivest

**Affiliations:** Neuroscience laboratory, CHU de Québec Research Center and Department of Molecular Medicine, Faculty of Medicine, Laval University, 2705 Laurier boulevard, Québec City, QC G1V 4G2 Canada

**Keywords:** mCSF, TREM2, β-Catenin, IL-34, Microglia, Innate immunity, Amyloid, Phagocytosis, Cognitive decline

## Abstract

**Background:**

Alzheimer’s disease (AD) is a progressive neurodegenerative disorder and the most common form of dementia in the world. Microglia are the innate immune cells of CNS; their proliferation, activation, and survival in pathologic and healthy brain have previously been shown to be highly dependent on CSF1R.

**Methods:**

Here, we investigate the impact of such receptor on AD etiology and microglia. We deleted CSF1R using Cre/Lox system; the knockout (KO) is restricted to microglia in the APP/PS1 mouse model. We induced the knockout at 3 months old, before plaque formation, and evaluated both 6- and 8-month-old groups of mice.

**Results:**

Our findings demonstrated that CSF1R KO did not impair microglial survival and proliferation at 6 and 8 months of age in APP cKO compared to their littermate-control groups APP_Swe/PS1_. We have also shown that cognitive decline is delayed in CSF1R-deleted mice. Ameliorations of AD etiology are associated with a decrease in plaque volume in the cortex and hippocampus area. A compensating system seems to take place following the knockout, since TREM2/β-Catenin and IL-34 expression are significantly increased. Such a compensatory mechanism may promote microglial survival and phagocytosis of Aβ in the brain.

**Conclusions:**

Our results provide new insights on the role of CSF1R in microglia and how it interacts with the TREM2/β-Catenin and IL-34 system to clear Aβ and ameliorates the physiopathology of AD.

## Background

Alzheimer’s disease (AD) is a progressive neurodegenerative disorder and the most common form of dementia in the world. The number of new cases is growing daily due to a lack of reliable biomarkers for diagnosis and efficient treatments [1]. Beta-amyloid (Aβ) is the hallmark of AD. Aβ aggregation leads to the formation of senile plaques. The cleavage of amyloid protein precursor (APP) by BACE1 and the enzymatic complex γ-secretase generates Aβ resulting in two main isoforms Aβ_1–40_ (Aβ_40_) and Aβ_1–42_ (Aβ_42_), the latter being the most toxic form [2]. Four to 6% of AD patients have genetic predisposition; early AD is associated with APP, PS1, and PS2 gene mutation, whereas late AD is related to a mutation of APOE 4 [3, 4].

Aβ deposits activate microglia, the innate immune cells of the central nervous system (CNS). Amyloid-exposed microglia synthetize tumor necrosis factor (TNF), which participates to the recruitment microglia and worsen inflammatory response [5]. Microglia originate from the embryonic yolk sac and migrate to the brain between embryonic days 8.5 and 10 in mice [6]. In adult CNS, microglia serve as brain macrophages, although they are distinct from other resident macrophages. Microglial cells dynamically survey the environment; they are responsible for the elimination of pathogens, cellular debris, dead cells, remodeling synapses, and the clearance of toxic proteins [7]. They are partly dependent on colony-stimulating factor-1 receptor (CSF1R) signaling for their maintenance, activation, proliferation, and self-renewal [8, 9].

CSF1R belongs to tyrosine kinase receptor family. It is broadly expressed in the organism by monocytes, macrophages, osteoclasts, and microglia [10]. CSF1R can bind two ligands namely macrophage-colony stimulating factor (mCSF) and interleukin-34 (IL-34). They have different binding sites, but their roles are quite equivalent [11]. In the brain, mCSF is primarily expressed by astrocytes, oligodendrocytes, and microglia, whereas IL-34 is mainly expressed by neurons [12]. Previous studies have demonstrated that administration of mCSF can generate macrophages from bone-marrow precursor cells and a substantial increase in the number of blood monocytes [13–15]. Blood levels of mCSF and other hematopoietic cytokines were found to decrease in AD patients, which was proposed as a good predictor for a rapid evolution from mild cognitive impairment pre-symptomatic to dementia [16]. In 2009, Boissonneault and colleagues have performed a study using multiple systemic mCSF injections in APP_Swe/PS1_ mice, and they reported a powerful effect of such a treatment to prevent the cognitive decline even at a critical age of the disease. MCSF-treated mice presented a lower number of Aβ plaques, which were associated with an increased number of microglial cells in the brain. The cytokine stimulates the clearance of Aβ_42_ by microglia and infiltrating bone marrow-derived cells [17]. In line with these findings, hippocampal injection of mCSF in AD mouse model induced the differentiation of bone marrow cells into bone marrow-derived microglia (BMDM), which resulted in improved cognitive decline [18].

IL-34, triggering receptor expressed on myeloid cells 2 (TREM2), and its adaptor DNAX-activating protein of 12 KDa (DAP12) have also an important role in the pathology. IL34 stimulates proliferation of monocytes and macrophages by binding CSF1R. Mizuno et al. have demonstrated that IL-34 injections ameliorated cognitive decline and reduced Aβ burden by upregulating insulin-degrading enzyme (IDE) in a mouse model of AD [19]. TREM2/DAP12 has a critical role in microglia survival, proliferation, and their ability to phagocyte Aβ [20]. A significant increase in TREM2 expression was found in plaque-associated microglia in AD patients [21–23]. A mutation leading to a loss-of-function in the TREM2 gene could decrease microglia survival and proliferation and a greater Aβ burden [24]. Indeed, experiments have shown that TREM2 deficiency led to an impaired phagocytosis of Aβ by microglia in a mouse model of AD [25]. β-Catenin has emerged as a crucial molecule for several pathologies [26–29]. CSF1R/TREM2/β-Catenin are linked by Src tyrosine kinase, the principal effector of CSF1R signaling; it can phosphorylate DAP12. DAP12 downstream intermediate molecule Pyk2 promotes stabilization of β-Catenin [23, 30]. In AD, Wnt/β-Catenin is downregulated while Wnt antagonist Dkk1 is upregulated [26]. Some research groups have reported that restoring β-Catenin in adult hippocampus was able to reverse synaptic loss, whereas administration of Dkk1 provokes a decrease in β-Catenin, TCF, LEF, and PSD-95 protein levels [31, 32].

Altogether, these data suggest that CSF1R/TREM2/β-Catenin and IL-34 pathways are important to prevent cognitive impairment in AD. In this study, we have investigated the role of CSF1R in a mouse model of AD using a conditional and specific microglial knockout (KO). We have found that CSF1R gene deletion failed to affect microglia survival and activation probably due to several compensatory mechanisms. The KO caused a significant increase in IL-34, TREM2, and β-Catenin gene expression together with the improvement of cognitive decline and a decrease in Aβ burden. These molecules together may compensate for the suppression of CSF1R in microglia.

## Methods

The aim of this study is to compare APP _Swe/PS1_ to APP CSF1R^−/−^ to understand the role of CSF1R in Alzheimer’s disease.

### Animals

We used only males for this study.

Conditional CSF1R knockout mice CSF1R-lox/CX3CR1-Cre/ER (called, cKO) B6.Cg-CSF1R *tm1jwp*/J mice (JaxMice; stock number 02212) were crossed with the B6.129-Cx3cr1tm2.1 (CreER)Jung/Orl mice (EMMA mouse respiratory; EM:06350). The resulting mouse has a tamoxifen-inducible CRE activity specifically in microglial cells, leading to a non-functional CSF1R protein. Mice were injected with tamoxifen at 10 weeks old.

Rosa^redTm^-CSF1R-lox/CX3CR1-Cre/ER mice: We crossed B6.Cg-Gt(ROSA)26Sor^tm14(CAG-tdTomato)Hze^ (JaxMice cat# 007914) mice with CSF1R-lox/CX3CR1-Cre/ER to confirm the deletion of CSF1R. Mice were injected with tamoxifen at 10 weeks old for 4 consecutive days.

APP_Swe/PS1_ male transgenic mice bearing a chimeric human/mouse β-amyloid precursor protein (APP_Swe_) gene and the human presenelin 1 gene (A246E variant). These mice were purchased from The Jackson Laboratory [Strain: B6C3-Tg (APP695)3Dbo Tg(PSEN1)5Dbo/J] and maintained on a C57BL/6 J background.

cKO mice and APP_Swe/PS1_ mice were crossed to obtain an APP_Swe/PS1_-CSF1R-lox; CX3CR1-Cre/ER (called, APP cKO). The resulting mouse has a tamoxifen-inducible CRE activity specifically in microglial cells, leading to a non-functional CSF1R protein. Mice were injected with tamoxifen at 3 months old.

All animals were acclimated to standard laboratory conditions (14 h light, 10 h dark cycle; lights on at 06:00 and off at 20:00 h) with free access to rodent chow and water. Protocols were conducted according to the Canadian Council on Animal Care guidelines, administered by the Laval University Animal Welfare Committee.

### Tamoxifen preparation and administration

Tamoxifen was dissolved in corn oil (Sigma-Aldrich cat#C8267) and ethanol 100% for 1 h at 37 °C, vortexed every 15 min. We used ∼ 75 mg tamoxifen/kg body weight, and 100 μl tamoxifen/corn oil solution was administered via intraperitoneal injection for 4 consecutive days.

### Sacrifices

All mice were deeply anesthetized with ketamine/xylazine (90:10) and sacrificed via intracardiac perfusion with 0.9% saline. Brains were retrieved and post-fixed 24 h in 4% PFA pH 7.4 and transferred in 4% PFA pH 7.4 + 20% sucrose for a minimum of 15 h. Brains were sliced in coronal sections of 20-μm thickness with a freezing microtome (Leica Microsystems), serially collected in anti-freeze solution, and kept at − 20 C until usage.

### Western blot

Brain protein lysate was extracted and quantified as previously described [33]. Proteins were loaded in 8–16% agarose precast gels (BioRad) and electroblotted onto 0.45 μm Immibilon PVDF membranes. Membranes were incubated with primary antibodies anti-β Catenin (Cell signaling rabbit 1:1000 cat#9562), TREM2 (R&D sheep 1:1000 cat#AF-1729), BDNF (Millipore rabbit 1:1000 cat#AB-1534), Syndecan-1 (Abcam mouse 1:1000 cat#AB-34164), IL-34 (R&D sheep 1:1000 cat#AF-5195), PSD95 (Millipore mouse 1:1000 cat#MAB-1596), BACE1 (Cerderlane rabbit 1:1000 AB-108394), APC (Novus biological rabbit 1:1000 cat#NBP2-15422), ABCB1 (Novus biological rabbit 1:1000 cat#NB-100-80870), and SMI 312 (Biolegend mouse 1:1000 cat#837904) followed by the appropriate horseradish peroxidase (HRP)-conjugated secondary antibodies and revealed by clarity (ECL) substrate (BioRad). Quantification was done by determining the integrative density of bands using Thermo Scientific Pierce by Image Analysis software v2.0. Optical values were normalized over actin.

### Immunofluorescent staining

Brain sections were washed (4 × 5 min) in KPBS then blocked in KPBS containing 1% BSA and 1% Triton X-100 (Sigma-Aldrich cat#T8787). Tissues were incubated overnight 4 °C with primary antibodies Iba1 (Wako rabbit 1:1000 cat#09-19741), 6E10 (Biolegend mouse 1:1000 cat#SIG-39320), CD31 (Ebioscience rat 1:1000 cat#11-0311-81). After washing sections in KPBS (4 × 5 min), tissues were incubated with the appropriate secondary antibodies for 2 h at room temperature. Followed by washes in KPBS (4 × 5 min), tissues were incubated 10 min with DAPI (Molecular Probes 1:10000 cat#D3571). After washes in KPBS (4 × 5 min) sections were mounted onto MicroSlides Superforst® (Fisherbrand cat#22-037-246) and cover slipped (Globe scientific cat#1419-10) with Fluoromount-G (Electron microscopy sciences cat#17984-25).

### Immunohistochemistry

Brain sections were washed (4 × 5 min) in KPBS then blocked in KPBS containing 1% BSA and 1% Triton X-100 (Sigma-Aldrich cat#T8787). Tissues were incubated overnight 4 °C with primary antibodies CSF1R (R&D sheep 1:750 cat#AF-3818). After washing sections in KPBS (4 × 5 min), tissues were incubated with the appropriate secondary antibodies for 2 h at room temperature. Followed by washes in KPBS (4 × 5 min), tissues were incubated with ABC mix (Vector Laboratories Vectasin® Elite ABC-HPR Kit, Peroxidase (Standard) 2.25 μl/ml sol A and 2.5 μl/ml sol B cat#PK-6100) for 1 h at room temperature. After incubation sections were washed in KPBS (4 × 5 min) and incubated up to 15 min with a DAB solution (Sigma-Aldrich H_2_O_2_ 0.003% cat#H1009 and Sigma-Aldrich DAB 0.05% cat#D7304-1SET), followed by washed in KPBS (4 × 5 min). Sections were mounted onto MicroSlides Superforst® (Fisherbrand cat#22-037-246) and cover slipped (Globe scientific cat#1419-10) after dehydration with DPX (Electron microscopy sciences cat#13510).

### Behavioral analyses

Novel object recognition task protocol [34] consists of 3 phases: habituation, familiarization, and test phase. The experimenter who observed and recorded the behavior was not aware of treatment and genotype of the tested animals (WT + vehicle, *n* = 10; APP_Swe_/PS1 + vehicle, *n* = 10; and APP + tamoxifen, *n* = 10). Baseline data were obtained at 3 months of age, whereas the effects of the treatment were determined at 6 or 8 months. Tests were made at 15 lx.

In nesting protocol [35], mice were placed 1 per cage at 3 months old. The experimenter who observed and recorded the behavior was not aware of treatment and genotype of the tested animals (WT + vehicle, *n* = 10; APP_Swe/PS1_ + vehicle, *n* = 10; and APP + tamoxifen, *n* = 10). After each NOR task, cages were cleaned a new pad was placed inside. After 24 h, the nests were analyzed and scored. The scores vary from 1 (untouched pad) to 5 (perfect nest).

### ELISA

Total brain homogenates were used for all the ELISA. For the Aβ_1–40_ (Millipore cat#EZHS40) we analyzed 50 μg per well following the protocol provided.

### Unbiased stereological count

Brains were serially sectioned as previously described and were stained with Iba1, 6E10, and DAPI as previously described. We counted microglia and plaques at × 20 magnification using an Axio Observer microscope equipped with Axiocam 503 monochrome camera and processed using with ZEN Imaging Software (Carl Zeiss Canada, Toronto, ON, Canada). Sampling characteristics for cortex sections are as follows: sampling interval 12, total number of sections 7, section sampling interval 3, and starting selection 1; for hippocampal sections: sampling interval 12, total number of sections 7, section sampling interval 2, and starting selection 1. After counting plaques using unbiased dissector, the mean plaque volume (MPV) was estimated using the rotator method. The estimated MPV was based on the length of line crossing each plaque using randomly oriented line. Four locations for each hippocampus and 3 for each cortex were sampled in 7 sections. Plaques and microglia were counted according to Gundersen unbiased counting rules, with optical fractionator method and sampling continued to a coefficient of error of 10% or less [36].

### CAA quantification

CAA was quantified with 6E10 staining (see staining procedure above). The number of amyloid-positive vessels into the cortex was manually counted over the whole brain 12 sections. The count was under blinded condition regarding the experimental groups [37, 38]. CAA frequency refers to the number of 6E10-positive vessels.

### Chimeric mice

Chimeric mice were generated to assess the infiltration of peripheral cells. They were generated at 1 month old using chemotherapy as previously described [39, 40]. APP_Swe/PS1_ and APP cKO received 80 mg/kg of busulfan administered i.p. every 12 h for 4 days, followed by 2 days of single i.p. injection of 100 mg/kg cyclophosphamide. After 24-h, 3 × 10^7^ bone marrow cells isolated from tibia and femur of donor mice (C57BL/6-Tg (CAG-EGFP) 10 sb/J JaxMice stock number 003291) were injected into tail vein of APP_Swe/PS1_ and APP cKO mice. They were more than 95% chimeric 7 weeks after bone marrow transplantation.

### Image acquisition

Image acquisition of fluorescent staining images was performed using a Zeiss LSM800 confocal microscope supported by the Zen software (2.3 system) using the 10×, 20×, and 40× lenses. Confocal images were then processed using Fiji (ImageJ Version 2.0.0-rc-43/1.51n). For analyses and bright field image acquisition of staining, Iba-1 and CSF1R, 8-bit grayscale TIFF images of the regions of interest were taken in a single sitting for whole protocols with a Qimaging camera (Qcapture program, version 2.9.10) attached to Nikon microscope (C-80) with the same gain/exposure settings for every image. To evaluate the level of Iba-1+ immune response in the regions of interest, the images were imported into ImageJ (1.37) and the percentage of area occupied by the staining was measured using the threshold parameter. Cell count was assesed manually using ImageJ (1.37). Analysis was performed in double blinded to avoid bias of analysis. Fluorescent staining of images was performed using a Zeiss LSM800 confocal microscope supported by the Zen software (2.6 system). Confocal images were then processed using Fiji (ImageJ Version 2.0.0-rc-43/1.51n).

### Statistical analyses and figure preparation

Data are presented as mean ± standard error of the mean (SEM). Statistical analyses were carried with the Prism software (version 8.0, GraphPad Software Inc.), comparing control groups vs. tested groups. Values were considered statistically significant if *p* < 0.05. All panels were assembled using Adobe Photoshop CC 2018 (version 19.1.0) and Adobe Illustrator CC 2018 (version 23.0.1).

## Results

### CSF1R is solely deleted in microglia

**Fig. 1:**
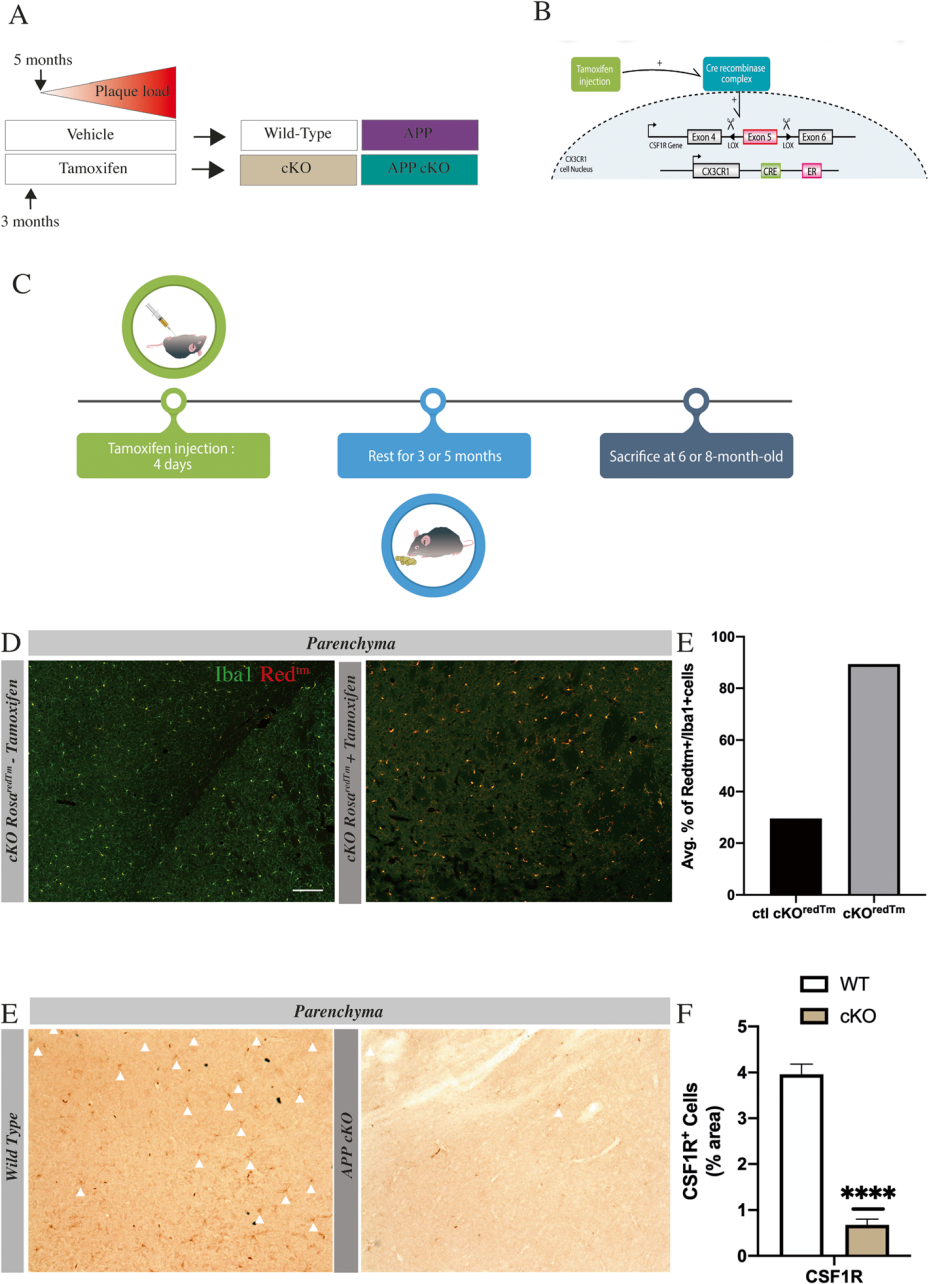
CSF1R deletion is solely deleted in microglia. **a** Experimental design. **b** Genetic construction of Cre/Lox system. **c** Timeline starting at 3 months. **d** Representative image of microglia (Iba1 green, Cre-recombination reporter gene Red^tm^) in WT and cKO. Scale bar = 200 μm. **e** Quantification of the average percentage of Red^tm+^/Iba1^+^ cells in Rosa^redTm^-CSF1R-lox/CX3CR1-Cre/ER without tamoxifen (ctl cKO^redtm^) or with tamoxifen (cKO^redtm^) *n* = 4 in each group. **e** Representative image of CSF1R staining in immunohistochemistry using 3-month-old WT and cKO mice. Scale bar = 200 μm. **f** Analysis of CSF1R staining at 3-month-old WT and cKO mice *n* = 4 in each group. (****p < 0.0001)

CSF1R conditional knockout (cKO) was made by a Cre/Lox system (Fig. 1b). Cre recombinase is under the CX3CR1 promoter. Lox sites flank CSF1R Exon 5. When tamoxifen is injected, the Cre recombinase complex translocates to the nucleus to interact with lox sites thereby CSF1R gene is non-functional. Mice were injected with tamoxifen at 3 months old (Fig. 1a). To determine whether the KO was efficient, we used Rosa^redTm^-CSF1R-lox/CX3CR1-Cre/ER mice. Mice express robust red^tm^ fluorescence following Cre-mediated recombination in CX3CR1 cells in the brain, meaning the knockout is effective (Fig. 1c, d). Quantifications show an endogenous activity of Cre-recombinase in mice without tamoxifen is around 20%; however, after induction, the knockout cells reach 89%. To confirm these results, we quantified by immunohistochemistry the percentage of CSF1R^+^ cells in the parenchyma of cKO mice. Results showed a robust decrease of CSF1R expression in cKO mice. These data indicate that our model is reliable and strongly efficient to induce the knockout selectively in microglia.

### The deletion of CSF1R does not affect microglia survival and delay cognitive decline in APP cKO mice

**Fig. 2:**
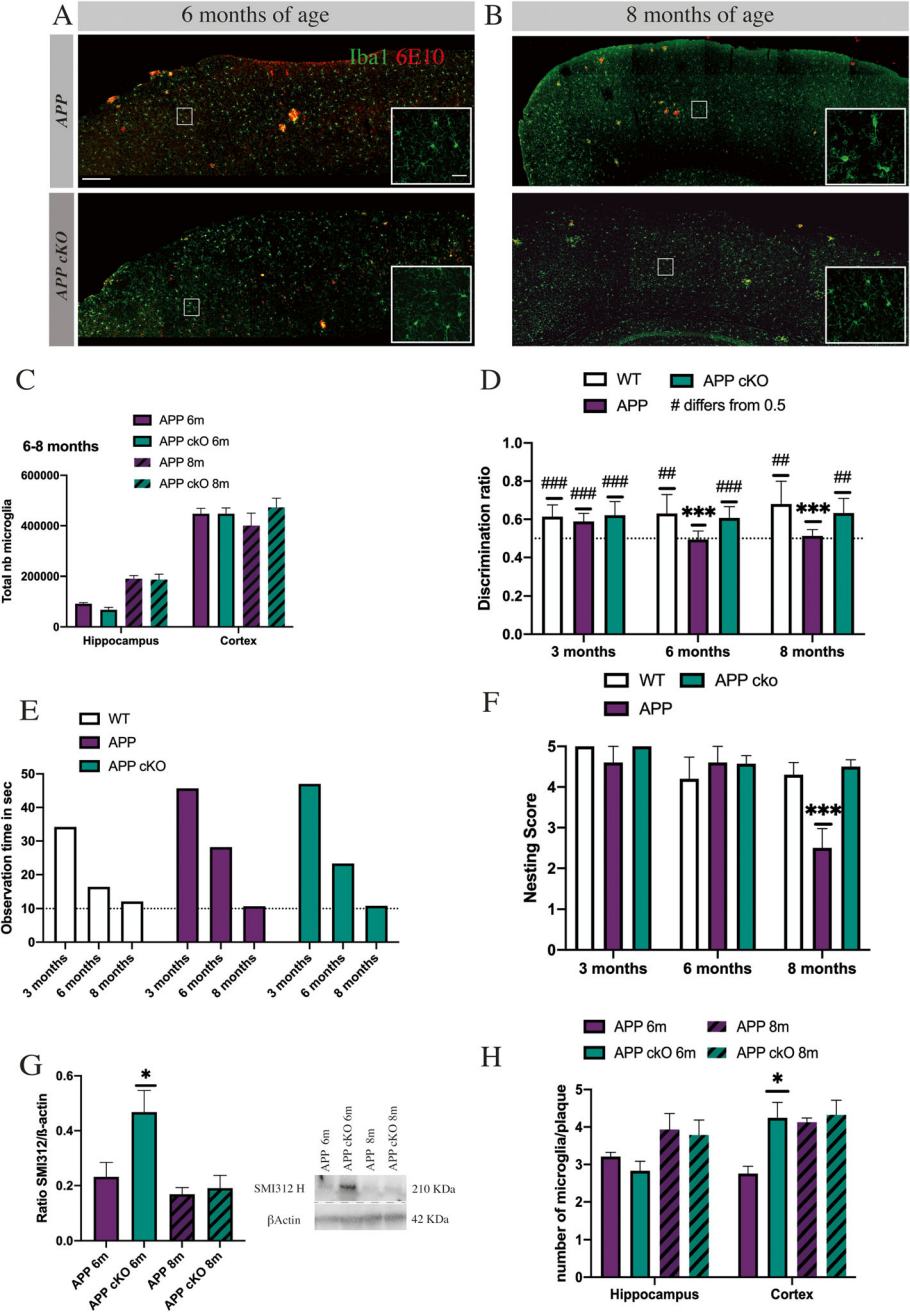
The deletion of CSF1R does not affect microglia survival and delay cognitive decline in APP cKO mice. **a**–**b** Representative image of microglia (Iba1 green) in APP_Swe/PS1_ (6–8 months old) and APP cKO (6–8 months old). Scale bar = 500 μm and 20 μm. **c** Unbiased stereological count of microglia in 6–8 months old APP_Swe/PS1_ and APP cKO. APP 6m, *n* = 6, APP 8m *n* = 4, *n* = 6 APP cKO 6m, and *n* = 4 APP cKO 8m. **d** NOR with 3-time points comparison of WT, APP_Swe/PS1_, and APP cKO from 3 to 8 months (^###^, ^##^ Discrimination differs from 0.5, APP_Swe/PS1_ 6-month-old ***p = 0.0002, 8-month-old ***p = 0.0007). *n* = 10 for all groups. **e** Average time of observation during NOR acquisition phase. **f** Nesting with 3-time points comparison of WT, APP_Swe/PS1_, and APP cKO from 3 to 8 months. Score range from 1 (untouched square) to 5 (perfect nest) (APP_Swe/PS1_ 8-month-old ***p = 0.0009). *n* = 10 in all groups. **g** Western blot analysis on 6- and 8-month-old APP cKO and their littermate controls. APP_Swe/PS1_ and APP cKO *n* = 7 (SMI312 heavy chain, *p = 0.0352). **h** Unbiased count of microglia per plaque in the hippocampus and cortex. APP_Swe/PS1_ and APP cKO *n* = 6 (cortex APP cKO 6 months *p = 0.0311)

As previously described, CSF1R is largely depleted in microglia. Here, we show, using an unbiased stereological analysis of Iba1-positive cells, no significative difference in microglia number into hippocampus and cortex at 6 and 8 months old in APP cKO compared to APP_Swe/PS1_ (Fig. 2a–c). These data are corroborating our previous findings, suggesting that KO has no impact on microglia survival in a specific model of neuronal injury [39]. To investigate the impact of the KO on cognition and the memory, APP cKO and their littermate controls were tested at 3, 6, and 8 months old using novel object recognition task (NOR). This test is a standard to evaluate the cognitive decline in the mouse model of AD [34]. The test measures the time spent on exploring the novel object compared to the common object. During the acquisition phase, every animal explored more than 10 s each object (Fig. 2e). The test phase showed no differences between groups at 3 months old. However, at 6 months old, APP_Swe/PS1_ mice present an expected sign of cognitive decline (***p = 0.0002), which was also confirmed at 8 months old (***p = 0.0007). APP_Swe/PS1_ mice spent equal time exploring both objects rather than exploring the novel one. Interestingly, APP cKO mice did not have the same cognitive decline at 6 or 8 months old. They spent an equivalent time to explore the novel object as the wild-type group did (Fig. 2d). Our data suggest a protective effect of CSF1R gene deletion to prevent cognitive decline in APP cKO mice.

The nesting behavior was conducted to evaluate the effect of CSF1R knockout on social withdrawal and apathy linked to AD. As depicted by Fig. 2f, the nest scores were equivalent between groups at 3 and 6 months old. However, 8-month-old APP_Swe/PS1_ mice had a lower score compared with WT and APP cKO groups (Fig. 2e, 2.5, APP_Swe/PS1_ ***p = 0.0009, 4.5, WT and 4.3 APP cKO). These data suggest that APP cKO does not exhibit a significant decrease in apathy and social withdrawal.

As behavioral tests unraveled a cognitive improvement, we assessed potential differences in microglia clustering and quantified dystrophic neurites as a measure of plaque toxicity (Fig. 2g, h). The integrity of neurofilament (NF) was measured using western blot, since NF protein expression levels provide information on axonal health [41]. Microglia clusters around plaques were quantified using unbiased stereology. NF expression levels are higher in 6-month-old APP cKO mice (*p = 0.0352) compared to APP at the same age. However, protein levels are similar at 8 months old in both groups of APP cKO and APP mice (Fig. 2g). Regarding microglia around plaques, their numbers are similar in the hippocampus in both groups of mice (Fig. 2h). On the other hand, their numbers are higher in the cortex of 6-month-old APP cKO mice than their littermate controls (4.24 and 2.75, *p = 0.0311). Such a difference was no longer found in 8-month-old mice. APP cKO mice exhibited roughly the same number of microglia per plaque at 6 and 8 months in the cortex area (4.24 and 4.3).

Altogether these results indicated that APP cKO mice do not present memory and social behavior impairment associated with AD. NOR and nesting tests did not show a significative difference between APP cKO mice and WT, unlike APP_Swe/PS1_ which, as expected, arbor a robust cognitive decline. Moreover, the number of microglia was equal between APP cKO and their control APP_Swe/PS1_, suggesting CSF1R is not the only receptor involved in microglia proliferation and survival in this model. The data regarding NF expression levels indicate that NF degradation is delayed following the KO.

### Long-term knockout reduces volume and plaque number, with the onset of cerebral amyloid angiopathy (CAA)

**Fig. 3:**
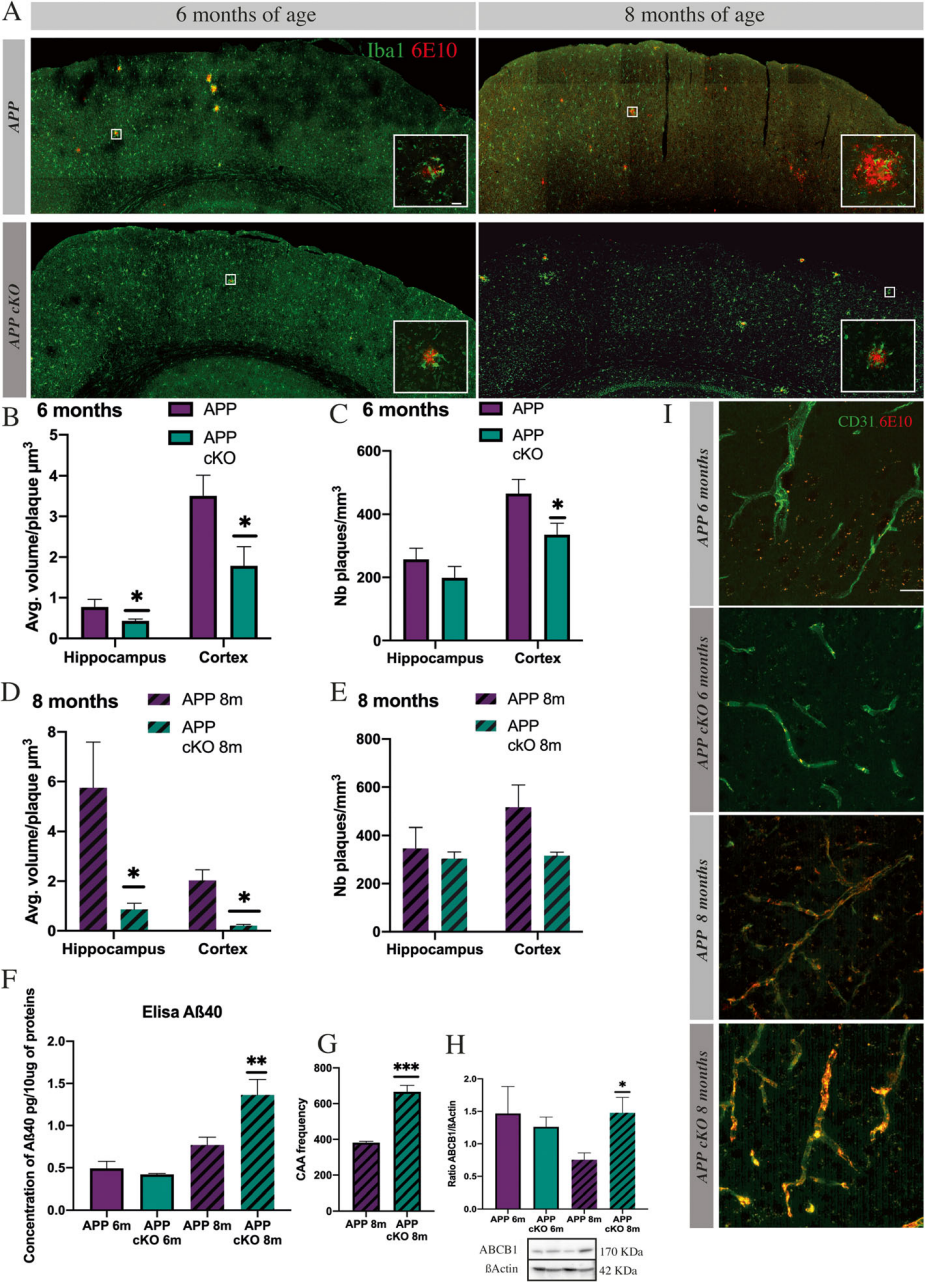
Long-term knockout reduces volume and plaque number, accompanied by cerebral amyloid angiopathy (CAA) onset. **a** Hemisphere stitches microglia (Iba1 green) amyloid plaque (6E10 red) in 6- and 8-month-old APP_Swe/PS1_ and APP cKO, scale bar = 500 μm. **b**–**e** Unbiased stereological analysis of plaques in the hippocampus and cortex. Analysis has shown a reduction in plaque volume at 6 (hippocampus *p = 0.0354, cortex *p = 0.0479) and at 8 months (hippocampus *p = 0.0320, cortex *p = 0.0227) in APP cKO. A reduction in the number of plaque/mm^3^ in the cortex (*p = 0.0270). APP_Swe/PS1_ 6m, *n* = 6, *n* = 6 for APP cKO 6m. **f** Elisa Aβ_40_ APP cKO 6 and 8 months old and their controls. APP_Swe/PS1_ (6–8m) *n* = 6 and APP cKO (6–8m) *n* = 7 (**p = 0.0098). **g** Frequency of CAA in APP_Swe/PS1_ and APP cKO at 8 months old, *n* = 4 (***p = 0.0002). **h** Western blot analysis for ABCB1 between APP cKO (6–8m) and their control. APP_Swe/PS1_ (6–8m) *n* = 6, *n* = 7 APP cKO (6–8m) (*p = 0.023). **i** Representative images of amyloid (6E10 red) on blood vessels (CD31 green) in APP_Swe/PS1_ and APP cKO at 6 and 8 months old. Scale bar = 50 μm

Cognitive impairment is correlated with the onset of Aβ plaque formation in the cortex and hippocampus in APP_Swe/PS1_. In this animal model, plaques are established and observable at 6 months old. Since CSF1R-deleted mice performed as well as WT mice, they should have fewer amyloid deposits in the hippocampus and cortex compared to APP mice. Sections were stained with Iba1 and anti-Aβ (6E10) at 6 and 8 months old (Fig. 3a). Here, we observed a difference in plaque number and volume in the cortex area. Using unbiased stereology, we quantified the number of plaques per region and the volume of Aβ deposit in APP cKO at 6 and 8 months old and their littermate controls (Fig. 3b–e). We observed a diminution by 2.1-fold of plaque volume in the APP cKO group at 6 months old (*p = 0.0354 hippocampus, *p = 0.0479 cortex). We also wanted to see if the number of plaques in each structure was changed. For this matter, we counted every plaque in both the hippocampus and cortex and then normalized the data with the volume of these structures. Results are expressed in the number of plaques per mm^3^, the software gave an unbiased count on the whole brain. The relative number of plaques is significantly decreased by 1.8-fold in the cortex of the APP cKO 6-month-old group (*p = 0.0270). At 8 months old, the volume of plaques is also reduced in the hippocampus (*p = 0.0320) and cortex (*p = 0.0227) compared to their littermate controls, respectively, by 6.6-fold and 10-fold. Actually, the volume of plaque does not differ between 6 and 8 months in APP cKO. However, regarding the number of plaques in the hippocampus or cortex, it remains similar in both groups at 8 months old (Fig. 3e).

These data provide clear evidence that deletion of CSF1R prevents the accumulation and/or induces a better clearance of Aβ in the brain.

The equilibrium between Aβ burden in the parenchymal and blood vessels is well described [42]. AD patients present a diminution of the transporter ABCB1 that impairs the efflux of Aβ in blood vessels. According to previous data, we studied the vascular amyloid to see if this transport was maintained. We used ELISA kit to quantify Aβ_40_ level in blood vessels. We did not observe any difference at 6 months in the APP_Swe/PS1_ or APP cKO groups. However, at 8 months old, we detected and significant augmentation of Aβ_40_ in the APP cKO group (**p = 0.0098) (Fig. 3f) associated with a stable expression of ABCB1 at 8 months for APP cKO (*p = 0.023) (Fig. 3h). These findings are corroborated by the count of 6E10-positive blood vessels in the cortex area (Fig. 3g, i) in both APP_Swe/PS1_ and APP cKO at 8 months old. CAA frequency is increased by 1.7-fold in the APP cKO group (***p = 0.0002). The CAA onset associated with the stable expression of ABCB1 indicates that CSF1R could play a role in CAA or at least it may contribute to downregulate ABCB1. These results also show that the peripheral immune system is not adequately activated to clear vascular amyloid.

Altogether, these data indicate that the KO has a beneficial effect in this model on cerebral amyloid load by decreasing the volume of senile plaques (Fig. 3b, e) and may accelerate vascular Aβ deposits and CAA (Fig. 3f–h).

### TREM2/β-Catenin and IL-34 brain protein levels following CSF1R gene deletion

We have previously demonstrated that a genetic ablation of CSF1R in a non-inflammatory model did not affect microglia proliferation and activation. Moreover, CSF1R knockout microglia overexpress TREM2 following nerve section. However, in the acute inflammatory model such as cuprizone, microglia are unable to proliferate and activate properly [39], indicating that the role of CSF1R is dependent on the microglial environment. Here, we detected the same amount of microglia in APP_Swe/PS1_ and APP cKO, and microgliosis around plaques was detected in both groups (Figs. 2a and 3a). This suggests that the genetic deletion in this model did not impair microglial proliferation and survival that may depend on other factors or compensatory mechanisms due to the CSF1R gene deletion.

**Fig. 4:**
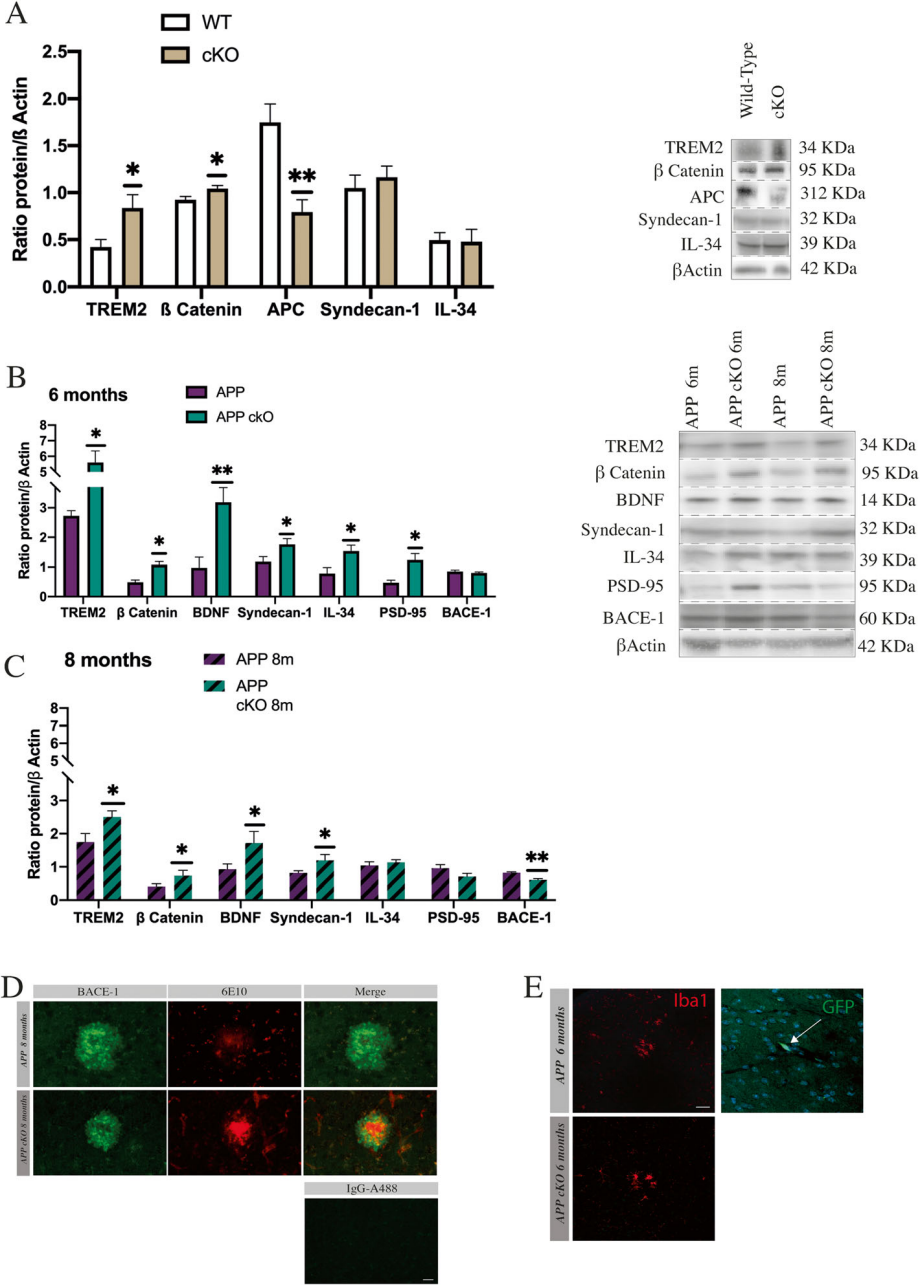
TREM2, β-Catenin, and IL-34 can compensate CSF1R deletion. **a** Western blot analysis of TREM2/β-Catenin pathway in WT and cKO at 3 months. WT, *n* = 6, *n* = 7 for cKO (TREM2, *p = 0.0384, β-Catenin **p = 0.0360, APC, **p = 0.0023, Syndecan-1, *p = 0.0498, IL-34, *p = 0.0274). **b** Western blot analysis on 6-month-old APP cKO and their littermate controls. APP_Swe/PS1_ and APP cKO 6-month-old, *n* = 6 (TREM2, *p = 0.0356, β-Catenin **p = 0.0027, BDNF, **p = 0.0071, Syndecan-1,*p = 0.0498, IL-34,*p = 0.0274, PSD-95,*p = 0.0120). **c** Western blot analysis on 8-month-old APP cKO and their littermate controls. APP_Swe/PS1_ and APP cKO 8-month-old, *n* = 6 (TREM2, *p = 0.0470, β-Catenin, *p = 0.0352, BDNF, *p = 0.0494, Syndecan-1, *p = 0.0378, Bace-1, **p = 0,0010). **d** Representative image of BACE-1 (green) and 6E10 (red) in APP_Swe/PS1_ and APP cKO 8-month-old. Scale bar = 50 μm. **e** Representative image of microglia in cortex area (red) and CX3CR1-GFP (green) in 6-month-old APP and APP cKO mice. CX3CR1 (green)-positive cell in a blood vessel in APP at  6 months. Scale bar = 100 μm

We then studied the expression of TREM2, β-Catenin, and IL-34 in young WT and cKO 10-week-old mice, because we postulated that these molecules can play an important role in AD by compensating for the KO. TREM2 protein levels increased by 2-fold in cKO mice compared to littermate controls (*p = 0.0384), which was associated with the stabilization of β-Catenin (*p = 0.0360) and the diminution of adenomatous polyposis coli (APC), a member of β-Catenin destruction complex (**p = 0.0023) (Fig. 4a).

We then looked at our both groups of 6- and 8-month-old mice and found a robust expression of TREM2, especially at 6-month-old APP cKO mice compared to APP_Swe/PS1_ age-matched animals (Fig. 4b) (*p = 0.036). This was again associated with an augmentation of β-Catenin protein levels (*p = 0.0027). Such upregulation of TREM2/β-Catenin may suggest that these pathways could compensate for the loss of CSF1R in order to keep microglia alive and functional. Interestingly, other very critical molecules increased in the 6-month-old cKO group, namely BDNF (**p = 0.0071), Syndecan-1 (*p = 0.0498), IL-34 (*p = 0.0274), and PSD-95 (*p = 0.0120) (Fig. 4b). These increases suggest a beneficial effect of CSF1R deletion on neurons, synapses, and microglia in the onset of the disease. A similar profile was found at 8 months old (Fig. 4c), at least for BDNF (*p = 0.0494) and syndecan-1 (*p = 0.0378), but not for PSD-95 and IL-34. It is noteworthy that BACE-1 significantly decreased (**p = 0.001) in the brain of 8-month-old cKO mice (Fig. 4c, d).

To further understand the implication of innate immunity in this model, we have generated chimeric mice with CX3CR1-GFP cells. With these chimeric mice, we were able to study whether systemic immune cells participate directly to the improvement of AD etiology by infiltrating the CNS. We sacrificed mice at 6 months old, and no CX3CR1-GFP-positive cells were observed into the brain of both the APP_Swe/PS1_ and APP cKO groups (Fig. 4e). These results are quite interesting since they suggest that beneficial effects of mCSF signaling inhibition on cognition and amyloid burden are mainly due to compensatory mechanisms in microglia and not via infiltrating monocytes.

### The number of microglia following knockout induction remains unchanged

**Fig. 5:**
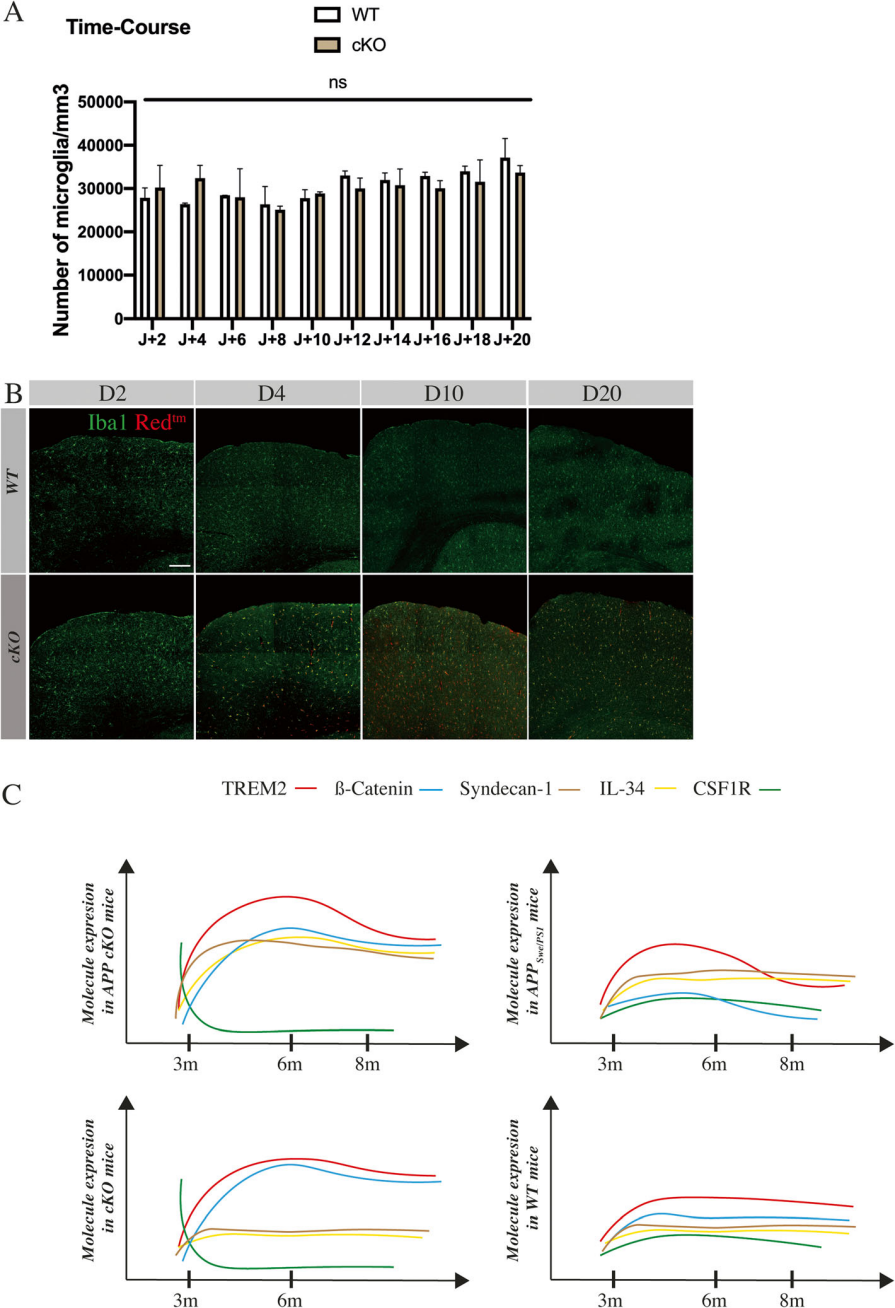
The knockout has no impact on microglia survival over the time. **a** Unbiased count of microglia in WT and cKO mice *n* = 20 for each group. **b** Representative image of microglia Iba^+^ (green) and knockout cells (Red^Tm^) for D2, D4, D10, and D20 after tamoxifen injections. Scale bar = 100 μm. *n* = 4 per group for each time point. **c** Fictive representation of molecular expression in APP cKO, APP_Swe/PS1_, cKO, and WT over the time

Several studies have shown a robust microglia lethality following pharmacological inhibition of CSF1R [43, 44]. We have previously demonstrated that the number of microglia in the hippocampus and cortex between APP_Swe/PS1_ and APP cKO is not statistically different (Fig. 2a). We aimed to show whether microglia following the KO died and repopulated the brain or signaling pathways previously described are sufficient to keep microglia alive. We have injected tamoxifen in WT and cKO mice at 3 months old every day for 4 days. We then have sacrificed mice every 2 days after the last injection, until 20th days post-injection. The count of microglia over the time indicates that microglia survived even depleted from CSF1R (Fig. 5a). Interestingly, at D4, we can observe that almost all microglia are KO, unlike at D2 suggesting that tamoxifen induces KO within 4 days after the last injection (Fig. 5b). These data strongly suggest that compensatory mechanisms must take place immediately following the conditional gene deletion to allow such microglial survival and activation in the brain of APP mice or CSF1R is far from being the only receptor involved in such process, at least in this mouse model of AD.

## Discussion

In this article, we aimed to study the role of CSF1R in AD. For this purpose, we have bred APP_Swe/PS1_ with CSF1R-lox/CX3CR1-Cre/ER mice. We induced the KO at 3 months old by injecting tamoxifen, before plaque formation, and we sacrificed animals at 6 or 8 months old. AD is a multifactorial disease in which CSF1R could play a major role. MCSF/CSF1R axis promotes microglial proliferation, survival, and activation [8]. In 2003, Mitrasinovic and colleagues have stimulated microglia with mCSF, and they found that the cytokine promoted phagocytosis of amyloid. In the same line, Boissonneault and colleagues found similar results by injecting mCSF in APP_Swe/PS1_ mice. CSF1R stimulation has beneficial effects on Aβ clearance by microglia and bone marrow-derived microglia (BMDM) [17]. However, growing evidence has shown recently that CSF1R inhibition could also prevent cognitive decline and amyloid deposition. Most of the research groups used a molecule that inhibits CSF1R signalization, namely PLX5622. Interestingly, by impeding mCSF signal, they observed a massive microglial depletion, suggesting a central role of mCSF/CSF1R axis in microglia survival [43, 45–49]. Lately, we have demonstrated the effect of CSF1R ablation on microglia is depending on the context. Actually, CSF1R-deleted microglia still survive and proliferate in a non-inflammatory environment, whereas they are unable to proliferate in a model of cuprizone-induced demyelination and inflammation [39].

In the present study, we used a Cre/Lox system to suppress CSF1R gene expression. The knockout affects 89% of microglia in the brain (Fig. 1d–f). Importantly, we observed a similar number of microglia following the KO (Fig. 5a) indicating that CSF1R is not essential for microglia to survive and proliferate in APP/PS1 mice (Fig. 2a–c). These findings are contradictory with studies using PLX5622, raising the question about the specificity of CSF1R inhibitor molecules or that the proposed compensatory mechanisms may not take place following the chemical inhibition of the receptor. TREM2 and β-Catenin may have a critical role to allow the survival and proliferation of microglia in the CSF1R cKO APP mice. TREM2 is an immunoreceptor expressed in the brain by microglia [23]; it activates survival pathway via β-Catenin [23, 25, 29, 50]. Moreover, the TREM2/β-Catenin pathway is important for microglial survival and proliferation [50]. Indeed, β-Catenin initiates the transcription of cyclin D1 and c-Myc. Interestingly, TREM2-deficient microglia have a reduced level of these controlling cell cycle molecules. In this model, these signaling pathways may compensate for CSF1R deletion. We observed that TREM2 increased by 2-fold in cKO mice compared to their littermate controls, and it is accompanied by the stabilization of β-Catenin since there is a diminution by 2.2-fold of APC (Fig. 4a). Adenomatous polyposis coli activation leads to β-Catenin degradation [51]. Clustering microglia around plaques may be linked to the formation of dystrophic neurites [52], although they may have a protective effect to the surrounding neurons [53]. TREM2 may be involved in these effects since TREM2-deficient microglia are less associated with plaques compared to their wild-type controls [54]. In our hands, TREM2 overexpression was not correlated with more microglia clustering around plaques (Fig. 2h). The number of Iba1^+^ per plaque remains similar between groups into the hippocampus, although we found more microglia per plaque in the cortex area of 6-month-old APP cKO. Remarkably, a very similar number of microglia per plaque was found in 6- and 8-month-old APP cKO mice. Microglia may then actively and more efficiently prevent the growth of amyloid plaques. A previous study in 5xFAD mice expressing human TREM2 exhibited a reduced number of dystrophic neurites in the cortex [55]. Here, we show that the marker of neurofilament SMI 312 is more expressed in 6-month-old APP cKO mice (Fig. 2g). However, at 8 months, all groups have a low level of SMI 312, which may correlate with dystrophic neurite formation. Results show a decrease in compensating pathways at 8 months old in APP cKO mice (Fig. 4b, c). This diminution could explain the delayed formation of dystrophic neurites in APP cKO.

These data strongly suggest that the TREM2/β-Catenin signaling pathway compensates for the CSF1R KO in this mouse model of AD. Such augmentation of TREM2 and stabilization of β-Catenin at 6 and 8 months of age suggest a positive effect on microglia proliferation and survival, which has been found to be beneficial in a mouse model of AD [56]. IL-34 and Syndecan-1 also increased in APP cKO, especially at 6 months of age, and IL34 was recently proposed to be the main brain factor to stimulate proliferation of microglia in the ME7 model of prion disease [57]. On interest, the elevation of IL-34 and Sydecan-1 was also found in CSF1R^op/op^ mice. Indeed, osteoclasts compensate by overexpressing IL-34 indicating a crosstalk or a similar role of both cytokines [58]. Furthermore, Mizuno and colleagues demonstrated that injections of IL-34 ameliorate cognitive decline and reduce Aβ burden in APP/PS1 mice [19]. Importantly, CSF1R-deficient mice present different phenotypes whether CSF1R is constitutively deleted or not [59]. Growing evidence shows that IL-34 signaling could be more important in adulthood than mCSF [60]. CSF1R is the main receptor of IL-34, but recently, alternative receptors have been proposed. Syndecan-1 is one of them. In vitro syndecan-1 is positively expressed when IL-34 binds CSF1R in macrophages [61]. In the APP cKO model, IL-34/syndecan-1 could be an alternative pathway for survival and proliferation of microglia. This hypothesis has recently been assessed in a model of chronic neurodegeneration by Obst and colleagues. They found that inhibition of IL-34 reduces microglial proliferation [57]. Overexpression of both TREM2/β-Catenin and IL-34/syndecan-1 is in line with other findings, corroborated by NOR and nesting test (Fig. 2d, e). NOR shows a delayed cognitive decline in APP cKO 6 and 8 months old associated with a decreased volume of plaques in the hippocampus and cortex (Fig. 3b, c). Microglia may be more efficient to phagocyte amyloid due to the overexpression of TREM2. These data together with the fact that BDNF increased by 3.3-fold in APP cKO in 6-month-old and 1.8-fold in 8-month-old mice clearly underline the beneficial outcome of the compensatory mechanisms (Fig. 4b, c). Of interest is the recent papers showing that TREM2 and β-Catenin upregulation is linked to microglia expressing BDNF [62, 63]. Finally, the inverse correlation of BACE-1 and TREM2 in AD cKO mice is in line with the overall beneficial property of the conditional CSF1R gene deletion. In this regard is the ability of Aβ_42_ to stimulate NF-kB pathway and BACE-1 gene transcription together with TREM2 inhibition [64].

To summarize molecular expression changes, we proposed a representative diagram in Fig. 5c.

Besides the positive effects of CSF1R deletion on AD, we have observed CAA onset in APP cKO mice at 8 months old. CAA is associated with AD in a large majority of cases [65]. ABCB1 transports Aβ_40_ from parenchyma to blood vessels and AD patients are known to present a lower expression level of ABCB1. This may allow the accumulation of Aβ in the brain [66–68]. Our data indicate that APP CSF1R KO mice at 8 months of age tend to have a greater accumulation of Aβ_40_ in blood vessels, due to a stable expression of ABCB1 compared to APP_Swe/PS1_ at the same age (Fig. 3f–h). β-Catenin has a critical role in blood-brain barrier (BBB) regulation; its over-expression may limit BBB damages and prevent CNS immune cell infiltration [69]. This may explain why cells of systemic origin failed to infiltrate the CNS in 6-month-old chimeric mice (Fig. 4e). β-Catenin is downregulated in AD, but ABCB1 expression is restored when this pathway is reactivated [70, 71]. According to these data, the over-expression of β-Catenin at 6 and 8 months old in APP cKO groups can maintain ABCB1 expression and therefore relieve the brain from Aβ burden. Blood monocytes are more efficient than microglia to clear vascular amyloid during the AD course, but the phagocytic capacity of monocytes in aging AD patients is greatly reduced [72, 73]. Consequently, the compensating pathways by resident microglia in APP cKO mice have clearly transient beneficial effects.

## Limitations

The first limitation is the use of one model of APP mice; this type of animal mimics the familial AD and not the sporadic form. Second, we observed an endogenous Cre/Lox activity before tamoxifen administration, meaning that the KO is naturally induced in some microglia. It could induce a cell adaptation. Third, we used only one type of KO; we could have compared the effects between two different KO, i.e., siRNA or antibody. However, these KO would have required more manipulation of animals and are less accurate.

## Conclusions

Our study aimed to understand the role of CSF1R in AD. With a conditional KO mouse model, we have induced CSF1R deletion at 3 months old, before plaque formation. Here, we demonstrated that strong compensatory pathways settled following the KO. Indeed, TREM2/β-Catenin and IL-34 expression increase leading to a reduction of plaque volume and a delayed cognitive decline. These ameliorations of mouse conditions are associated with the overexpression of molecules acting on maintenance and protection of neurons and synapses. We have also demonstrated that CSF1R deletion did not impair microglia proliferation and survival probably due to the compensating TREM2/β-Catenin and IL-34 pathways. These signaling pathways seem primordial for microglia and AD etiology when CSF1R gene is deleted in a conditional manner.

## Data Availability

All data generated or analyzed during this study are included in this published article.

## Abbreviations

Aβ: Amyloid-beta
AD: Alzheimer’s disease
APC: Adenomatous polyposis coli
APP: Amyloid precursor protein
cKO: Conditional knockout
CSF1R: Colony-stimulating factor-1 receptor
DAP12: DNAX-activating protein of 12 KDa
IL-34: Interleukin-34
KO: Knockout
mCSF: Macrophage colony-stimulating factor-1 receptor
NOR: Novel object recognition task
TNF: Tumor necrosis factor
TREM2: Triggering receptor expressed on myeloid cells 2
WT: Wild type
